# Formation of Phases in Reactively Sintered TiAl_3_ Alloy

**DOI:** 10.3390/molecules25081912

**Published:** 2020-04-21

**Authors:** Andrea Školáková, Pavel Salvetr, Jindřich Leitner, Tomáš Lovaši, Pavel Novák

**Affiliations:** 1 Department of Metals and Corrosion Engineering, University of Chemistry and Technology, Technická 5, 166 28 Prague 6, Czech Republic; pavel.salvetr@comtesfht.cz (P.S.); lovasit@vscht.cz (T.L.); panovak@vscht.cz (P.N.); 2 Institute of Physics, Academy of Sciences of the Czech Republic, Na Slovance 2, 182 21 Prague 8, Czech Republic; 3 COMTES FHT, Průmyslová 995, 334 41 Dobřany, Czech Republic; 4 Department of Solid State Engineering, University of Chemistry and Technology, Technická 5, 166 28 Prague 6, Czech Republic; leitnerj@vscht.cz

**Keywords:** Ti-Al system, reactive sintering, combustion, kinetics, microstructure

## Abstract

This work highlights new results on the synthesis of the TiAl_3_ intermetallic phase using self-propagating high-temperature synthesis. This method is considered a promising sintering route for intermetallic compounds. It was found that the reactions proceed in two stages. Below the melting point of aluminum, the Ti_2_Al_5_ phase forms at 450 °C after long annealing times by a direct solid-state reaction between the aluminum and titanium, and is converted consequently to TiAl_3_. This is a completely new finding; until now, many authors have believed in the preferential formation of the TiAl_3_ phase. The second stage, the self-propagating strongly exothermic reaction, proceeds above the melting point of aluminum. It leads to the formation of the TiAl_3_ phase accompanied by Ti_2_Al_5_ and Ti_3_Al phases. The reaction mechanism was shown in the form of chemical equations, which were supported by calculating Gibbs energy. Reaction temperatures (T_onset_, T_maximum_, and T_offset_) were determined after induction heating thanks to recording by an optical pyrometer. This finding provides completely new opportunities for the determination of activation energy at heating rates, in which common calorimeters are not able to detect a response or even measure. Now, the whole procedure will become accessible.

## 1. Introduction

Titanium aluminides belong to a group of modern materials that could replace nickel-based alloys in high-temperature applications such as in the aerospace industry. They offer low density, good high-temperature creep strength, stiffness, high melting points, and oxidation resistance [1,2]. However, their wider applications are hampered by low-temperature ductility [1]. They have been applied practically as exhaust valves, turbine superchargers, and low-temperature turbine blades in a GEnx^TM^ engine, which is the first commercial aircraft engine using Ti-Al alloy [1,3].

Equilibrium phases occurring in Ti-Al systems are Ti_3_Al-compounded with hexagonal close-packed superlattice structures and TiAl and TiAl_3_ intermetallic compounds with tetragonal structures [3,4]. Further, TiAl_2_ and Ti_2_Al_5_ phases are found on the aluminum-rich side of the diagram [3]. As can be seen, titanium-rich phases (Ti_3_Al and TiAl) exist over a large range of compositions, meanwhile the TiAl_3_ phase forms as a line compound only at [3]. The occurrence of phases depends on chemical composition and temperature. However, when alloys are prepared by methods like reactive sintering, this equilibrium phase diagram does not apply because the process is significantly unbalanced.

The reactive-sintering synthesis of intermetallics is usually connected with the evolution of large amounts of heat, which allows the reaction to be sustained and propagated without the need for additional heating. Therefore, this process is also called the self-propagating high-temperature synthesis (SHS). SHS lies in the rapid heating of the powder mixture, leading to the formation of intermetallic compounds as the reaction zone propagates through the sample [5,6]. The required temperature of heating is usually close to the melting point of aluminum in the case of the synthesis of aluminides [5]. The main advantages are the simplicity of the process, short-reaction time, saves energy, and the resulting purity of the products [7,8]. Moreover, reactants are in the form of fine powders, which are cold-pressed into the green body; thus, no expensive casting for a subsequent heat treatment is needed [9]. SHS can be performed in two ways. The first way involves heating only one side of the sample called the plane wave propagation (PWP). Meanwhile, the second one comprises heating whole powder mixtures, and this procedure is called a thermal explosion (TE) [10]. Both methods have their drawbacks. Precombustion reactions occur in slow methods of sintering, and they can modify the reaction mechanism. Fast-heating methods suffer from very poor reliability and reproducibility of the results [11].

Many investigators have studied the Ti-Al system, because the mechanism of the phases’ formation, specifically in the TiAl_3_ alloy composition, has not been explained yet. Moreover, the existing results are considerably different. So far, it is known that the reaction is initiated between solid titanium and liquid aluminum [5,12], and it starts approximately at the melting point of aluminum [5,6,8]. The initial phase, which forms preferentially, is considered the TiAl_3_ phase [5,8,13]. Its easy formation is associated with low, free energy compared with Ti_3_Al, TiAl, or Ti_2_Al_5_ phases [12] and kinetic preferences [14]. However, the real phases’ formation sequence is not known yet in the TiAl_3_ alloy. We focused on the chemical composition corresponding to the TiAl and Ti_3_Al phases in our previous studies [15,16], where the preferential formation of the Ti_2_Al_5_ phase was confirmed. Other work showed that the main reaction is associated with the formation of Ti_3_Al [11]. Therefore, it is necessary to observe certain reaction conditions to describe the sequences of the phases’ formations exactly.

Unfortunately, reactive sintering strongly depends on complex parameters [11], including properties of reactants, heating rates, time, and temperature of reactions. To control the process efficiently, reaction conditions have to be described. Heating rates belong to the parameters, which mainly affect the phase composition, microstructure, and reaction temperature. For this reason, the effect of the heating rate on reaction temperatures and phase compositions were studied in a TiAl63 alloy composition corresponding to the TiAl_3_ phase. The values of temperatures associated with the formation of phases could subsequently be used for thermodynamic calculations. The second part of this work was focused on the phases’ formation at temperatures lower than the melting point of aluminum.

## 2. Results

### 2.1. Thermal Analysis

**Figure 1:**
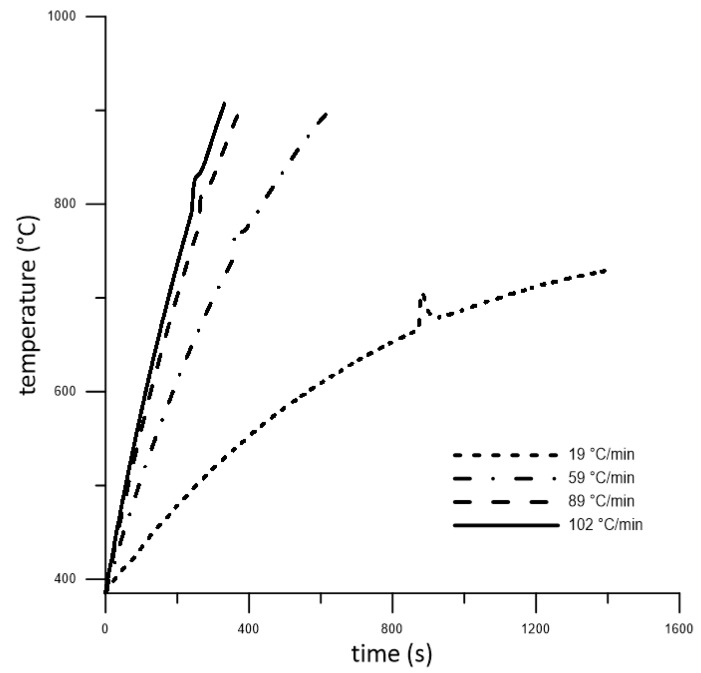
The heating curves obtained at various heating rates.

The heating rate is one of the most important parameters, whose effect is necessary to understand in order to be able to describe the reactions during reactive sintering. For this reason, cold-pressed powders were heated by various heating rates from the slowest to the fastest one, which can be achieved by induction heating (19–102 °C/min). The obtained curves are shown in Figure 1. According to the shape of the curves, it can be seen that only one exothermic peak was observed during heating. This peak was associated with the formation of phases. The reaction temperatures determined from the curves are listed in Table 1. As can be seen, all temperatures comprising the start of reaction (T_onset_) and the end of reaction (T_offset_), as well as the maximum achieved during heating, increased with an increased heating rate (Table 1). All exothermic reactions started at temperatures higher than the temperature of the melting point of aluminum. This means that the formation of phases took place on the interface between liquid aluminum and solid titanium. Only the heating rate of 19 °C/min caused the reaction to start at 662 °C, close to the melting point of aluminum (660 °C) and peritectic temperatures (665 °C) in diagram Ti-Al [17]. Therefore, this would suggest that TiAl_3_ phases formed after the peritectic reaction resulted in the presence of a liquid phase during heating at this heating rate. This claim was confirmed by studying the microstructure and XRD, as will be shown below.

Further, it can be seen that the exothermic peak is less noticeable as the heating rate increased (Figure 1). Phases formed during a very short time at heating rates 59–102 °C/min because the observed peaks were narrower. Various heating rates also allowed for the calculation of the activation energy of phases formation according to the Kissinger equation Equation (1) [18]. Its value was determined to be only 17 kJ/mol meaning there was a fast formation of phases and reaction between titanium and aluminum. This value was probably the sum of all reactions occurring during the observed exothermic peak. A similar result was observed in work [11] where the ignition energy was calculated.

(1) $$\ln\left(\frac{T^{2}}{\beta }\right)=\frac{E}{RT}+C$$

where T (K) is the temperature of the exothermic peak, β (K/min) is the heating rate, E (kJ/mol) is the activation energy, R (kJ/(K·mol)) is the universal gas constant, and C is the constant.

### 2.2. Phase Composition and Microstructure of Alloys Heated at Various Heating Rates

**Figure 2:**
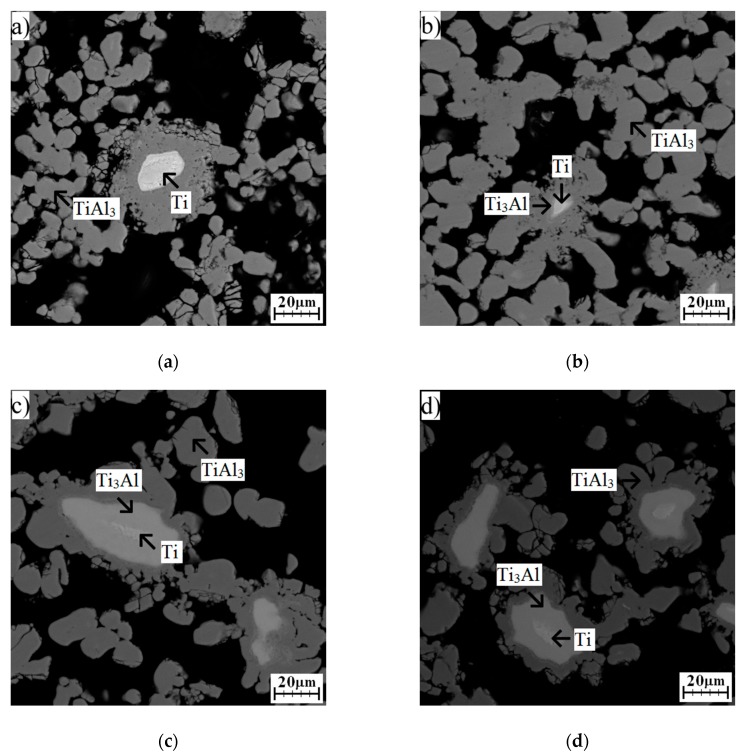
The microstructures obtained at various heating rates: (**a**) 19 °C/min, (**b**) 59 °C/min, (**c**) 89 °C/min, and (**d**) 102 °C/min.

**Figure 3:**
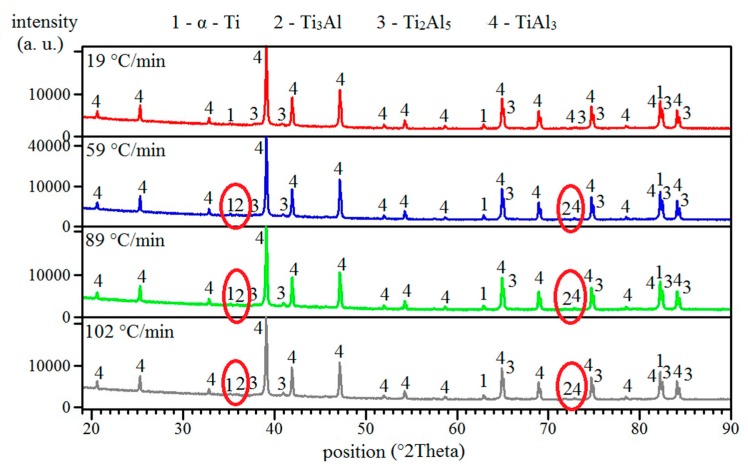
The XRD patterns of TiAl63 alloys obtained by various heating rates in the induction furnace.

The lowest heating rate caused the microstructure to consist of only unreacted particles of titanium surrounded by TiAl_3_ phases and pores (Figure 2a). The XRD analysis also revealed the presence of the Ti_2_Al_5_ phase (Figure 3). All aluminum reacted with the titanium, leading to the formation of an aluminum-rich phase. Thus, no aluminum was found (Figure 3). Typical positions belonging to peaks are identified for all phases. Major peaks, including minor peaks, are labeled by numbers. All phases are always identified according to the major peaks, and their presence must be confirmed by minor ones. For this reason, all peaks are marked. The changes in phase composition are always labeled by circles or arrows. A higher heating rate (59 °C/min) enabled the formation of the Ti_3_Al phase around unreacted particles of titanium (Figure 2b). This phase had to form after the reaction between titanium and the already present aluminum-rich phase, probably with TiAl_3_ or Ti_2_Al_5_ phases, or thanks to high temperatures, as will be shown in part 2.3. The TiAl_3_ phase formed as the main phase after annealing at 59 °C/min (Figure 2b). The Ti_2_Al_5_ phase was also found, and was detected by the XRD measurements (Figure 3). As the heating rate increased, the area of the Ti_3_Al phase was larger (Figure 2c,d). Phase composition remained identical (Figure 3). Results from the EDS analysis are shown in Table 2, and they are added for confirmation of the presence of the Ti_3_Al phase.

As can be seen, the TiAl_3_ phase was found during all applied heating rates; therefore, it can be assumed that the observed exothermic peak is associated mainly with its formation. The formation of this phase is, however, accompanied by the formation of other phases, namely, the Ti_2_Al_5_ and Ti_3_Al phases. The exothermic peak is thus affected by the released heat associated with the formation of these phases. It is also evident that the highest exothermic response was observed during heating at 19 °C/min when the Ti_3_Al phase was not detected (Figure 2a). The formation of aluminum rich-phases is much more exothermic at a low-heating rate. Significant porosity was also found, and it is a typical phenomenon for reactive sintering. All initiation temperatures were higher than the melting point of aluminum, but liquid aluminum did not fill pores sufficiently, probably due to a limited time of existence of the melt or its low fluidity. Another cause of high porosity from the obtained microstructure is connected with the different diffusivities of titanium and aluminum supporting the Kirkendall effect.

### 2.3. Formation of Phases in Samples Annealed at Temperatures Below Melting Point of Al

**Figure 4:**
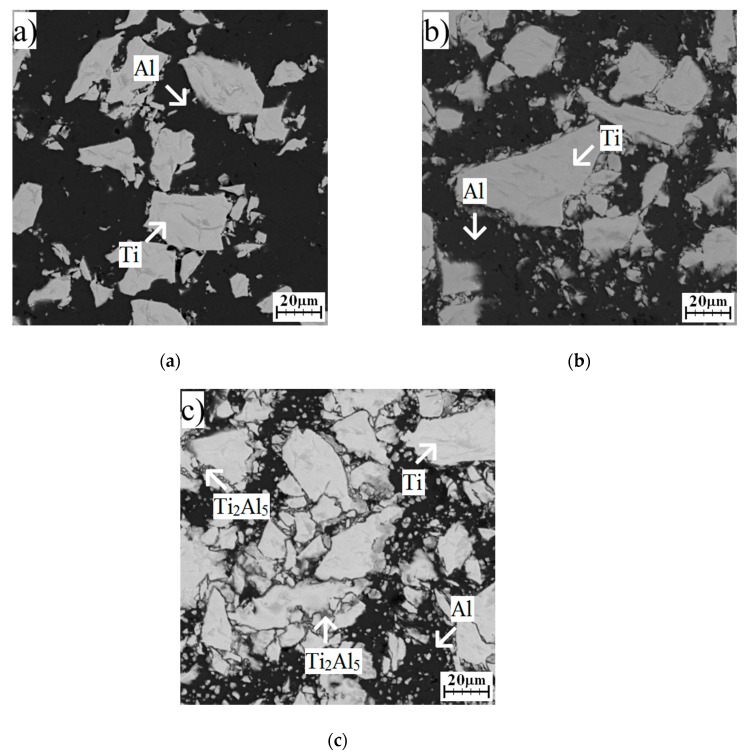
The microstructure of TiAl63 alloys annealed at 450 °C for (**a**) 8 h, (**b**) 24 h, and (**c**) 48 h.

**Figure 5:**
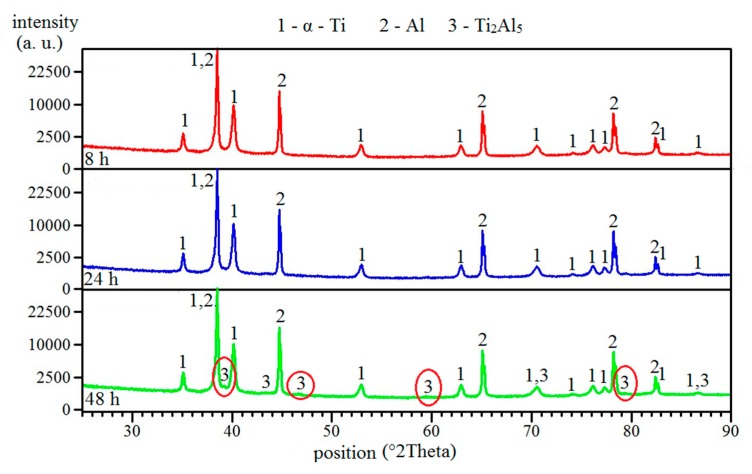
The XRD patterns of TiAl63 alloys annealed at 450 °C for 8, 24, and 48 h.

Annealing at 450 °C for 8 and 24 h did not cause the formation of any intermetallic phase (Figure 4a,b). Only unreacted particles, aluminum, and titanium were found, and no presence of intermetallic phases was detected by the XRD analysis (Figure 5). The Ti_2_Al_5_ phase formed after 48 h, and this one could be observed at the interface of aluminum and titanium (Figure 4c and Figure 5). Ti_2_Al_5_ formed due to the reaction between solid aluminum and solid titanium without the need of the melt as the transport medium. Thus, the whole reaction proceeded in solid state, and the formation had to be controlled by a mutual solid-state diffusion of aluminum and titanium. This situation is described by Equation (2). The values of Gibbs energy were calculated based on works [4,15].

2 Ti (s) + 5 Al (s) → Ti_2_Al_5_ (s) (ΔG = −223 kJ/mol) (2)

**Figure 6:**
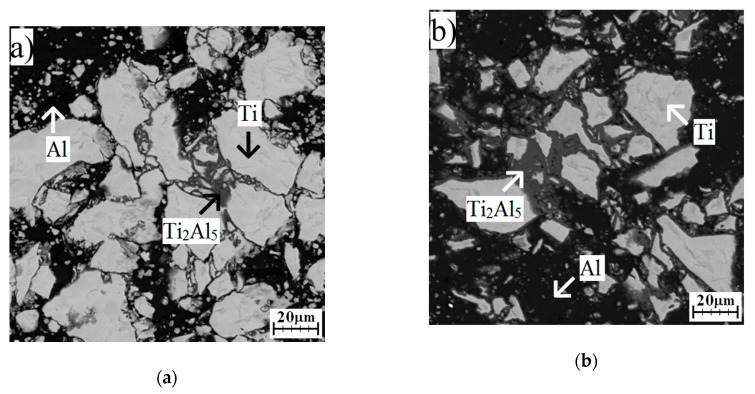

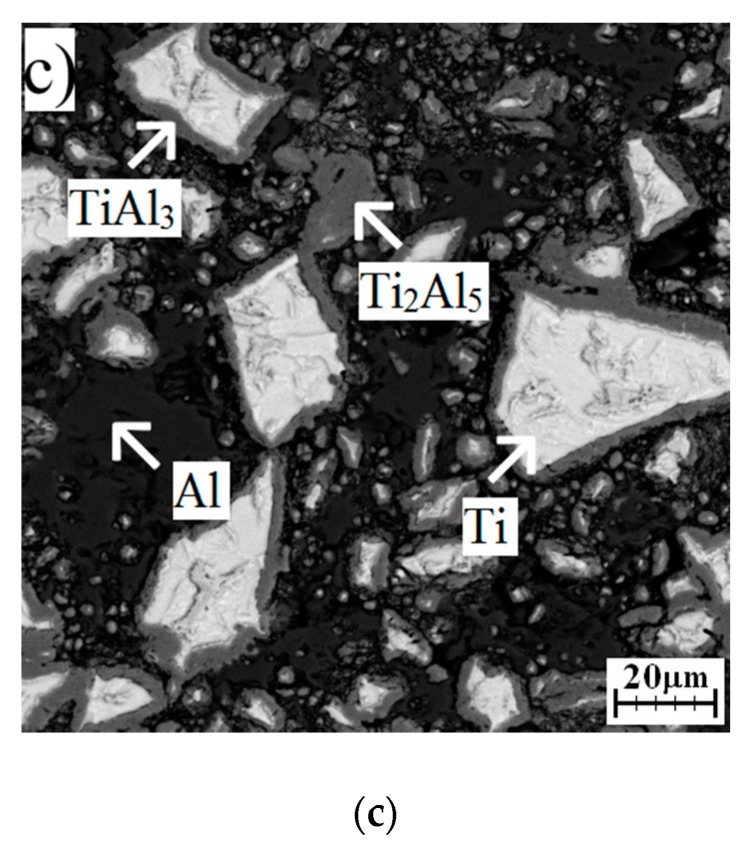
The microstructure of TiAl63 alloys annealed at 500 °C for (**a**) 8 h, (**b**) 24 h, and (**c**) 48 h.

**Figure 7:**
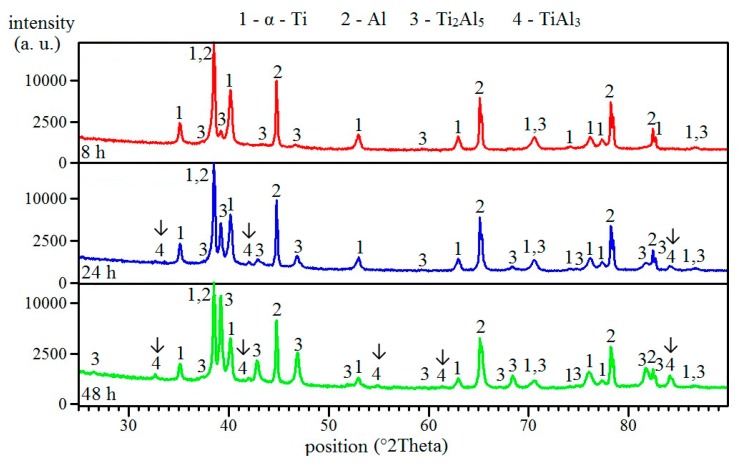
The XRD patterns of TiAl63 alloys annealed at 500 °C for 8, 24, and 48 h.

The Ti_2_Al_5_ phase was still found at the interface of aluminum and titanium after annealing at 500 °C for 8–48 h (Figure 6a–c), and its area was larger. The reaction was still in solid state, and other aluminum-rich phases formed. This phase was TiAl_3_, which was detected after annealing for 24 and 48 h, and only an XRD analysis proved its formation (Figure 7). It can be assumed that the TiAl_3_ phase formed thanks to the reaction between solid aluminum and an already present Ti_2_Al_5_ phase, as can be seen in Equation (3).

Ti_2_Al_5_ (s) + Al (s) → 2 TiAl_3_ (s) (ΔG = −27 kJ/mol) (3)

**Figure 8:**
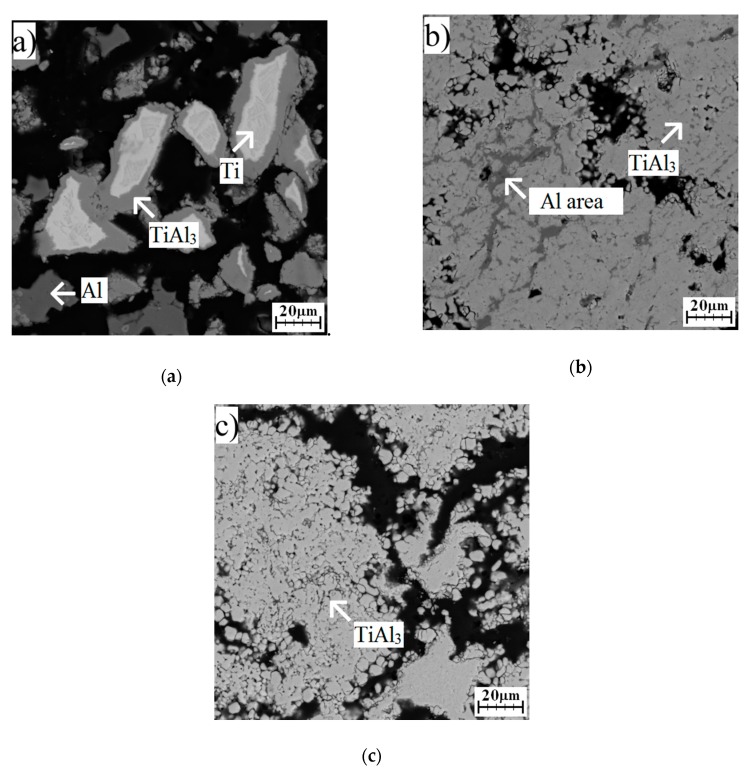
The microstructure of TiAl63 alloys annealed at 600 °C for (**a**) 8 h, (**b**) 24 h, and (**c**) 48 h.

**Figure 9:**
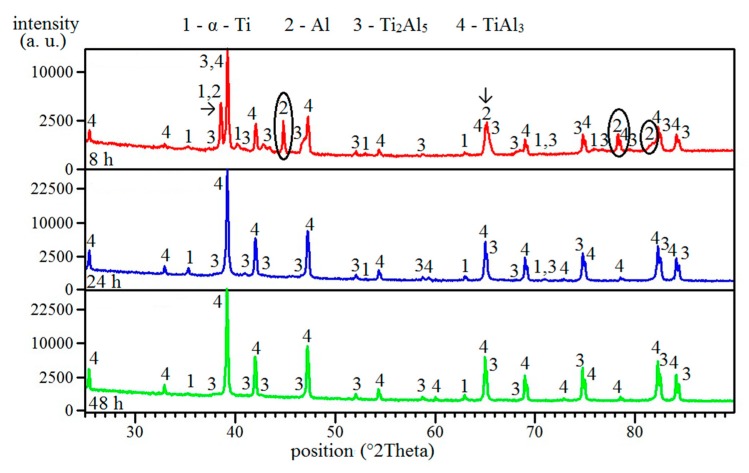
The XRD patterns of TiAl63 alloys annealed at 600 °C for 8, 24, and 48 h.

The TiAl_3_ phase replaced Ti_2_Al_5_ at the interface of aluminum and titanium after annealing at 600 °C for 8 h (Figure 8a). Aluminum was not detectable by XRD after annealing for 24 and 48 h (Figure 9), and the microstructure was much more reacted (Figure 8b,c). The structure consisted of the TiAl_3_ phase and the rest of the unreacted aluminum (Figure 8b). The chemical composition of this area is shown in Table 3. Residual unreacted aluminum was not detected by the XRD, probably because of its low amount. Only the TiAl_3_ phase was found in the structure annealed for 48 h (Figure 8c). XRD diffraction shows that the titanium and Ti_2_Al_5_ phase was still present locally (Figure 9).

## 3. Discussion

As can be seen, the formation of titanium aluminides is not so exothermic (Figure 1). However, induction heating is the most suitable for the sintering of these intermetallics, as was mentioned in work [19]. The used midfrequency induction heating has the capability to rapidly heat the whole sample volume, and it is used for the preheating of powder mixtures before the PWP-SHS reaction due to the low exothermicity of titanium aluminides [19]. However, we found induction heating also to be a suitable source for TE-SHS of TiAl_3_. Work [11] also studied the ignition of TiAl_3_. When we compared the obtained heating curves, our temperature profile did not show a plateau appearing at approximately 645 °C, which corresponds to the formation of the melt. This can be caused by the heat source when work [11] used a laser beam for combustion. Laser power (approximately 60 W) was focused on only one small spot, and this spot was observed. Moreover, their studied tablet of the sample was very thin. Our samples had a height of approximately 20–30 mm. Moreover, the reactions were probably initiated very quickly after the melt occurred, so the plateau was very short, practically invisible. The microstructure of the obtained samples contained unreacted titanium, and it is known that the dissolution of the higher-melting elements (titanium) in molten aluminum is one of the major kinetic aspects for combustion of aluminides [19]. The explanation lies in the particle size of titanium. The used particle size was 44 µm, which resulted in a very fast dissolution in molten aluminum and better interfacial contact between particles. Thus, the kinetic barrier could not arise. According to heating curves, the exothermic reaction took only a few seconds, during which intermetallic phases formed. Therefore, unreacted particles of titanium were observed in microstructures (Figure 2a–d). Unreacted particles of titanium were also observed in work [11], where the reaction was even faster. In order to obtain a single TiAl_3_ phase, the necessity of preheating the green bodies was also demonstrated [20]. Activation energy 17 kJ/mol was similar to the value set in work [11], but it was approximately twice smaller than the activation energy calculated for the TiAl_3_ phase in work [21], suggesting that our activation energy is probably a combination of the values for the formation of more phases. This statement is supported by the results of the XRD analysis showing the presence of Ti_3_Al, Ti_2_Al_5_, and TiAl_3_ phases after induction heating. The activation energy can also be affected by the surface quality of the powders. All reaction temperatures increased with an increased heating rate, which was shown in, e.g., [22]. On the basis of the obtained results, it could also be deduced that increasing the amount of aluminum caused the reaction temperatures to increase [22]. In high-aluminum alloy (TiAl63), much more melted aluminum forms during SHS reactions, which allows the dissolution of more titanium. The liquid phase, hence, acts as the transport medium for reactants, because the diffusion coefficients of metals in the liquid phase are several orders higher than in solid state. Thus, there are more places where the intensive reactions between titanium and aluminum proceed at the same time. The formation of intermetallic compounds is associated with the release of heat, which results in the shift of temperatures.

Many works claim that the first phase, which forms in the whole Ti-Al system at all compositions (Ti_3_Al, TiAl, and TiAl_3_), is the TiAl_3_ phase [8,14,21,23,24,25,26]. It is attributed to aluminum, which is considered as the dominant diffusing component through TiAl_3_, resulting in its easier formation [21]. However, we revealed the preferential formation of other aluminum-rich phases of Ti_2_Al_5_, which was confirmed in this work. This phase was observed in our previous works in low-aluminum alloy composition, Ti_3_Al [16], and equimolar alloy composition, TiAl [15]. It is in good agreement with the thermodynamic analysis stated in work [15]. The Ti_2_Al_5_ phase reacted with aluminum, and TiAl_3_ could have arisen because this phase appeared at the interface of Ti_2_Al_5_/Al. Work [15] also explained how Ti_3_Al can form in this alloy composition. Its existence is associated with Gibbs energy, which is negative in the temperature range 400–900 °C. This phase, thus, could form in high-aluminum alloys at high temperatures (above 735 °C), as was observed after induction heating. At lower temperatures, the reactions lead to the formation of high-aluminum phases, which are preferred in this alloy composition; therefore, the Ti_3_Al phase was not found during lower temperature annealing experiments in solid state. According to obtained results, it is obvious that microstructures after induction heating and after annealing at temperatures are different. At the temperature below the melting point of aluminum, the solid-state diffusion is the controlling mechanism; hence, a long period of time (8–48 h) is needed for the formation of detectable amounts of Ti_2_Al_5_ and TiAl_3_. During induction heating, the sample is heated quickly; hence, the solid-state reaction stage is skipped and the reactions are initiated at the solid–liquid interface.

## 4. Materials and Methods

**Figure 10:**
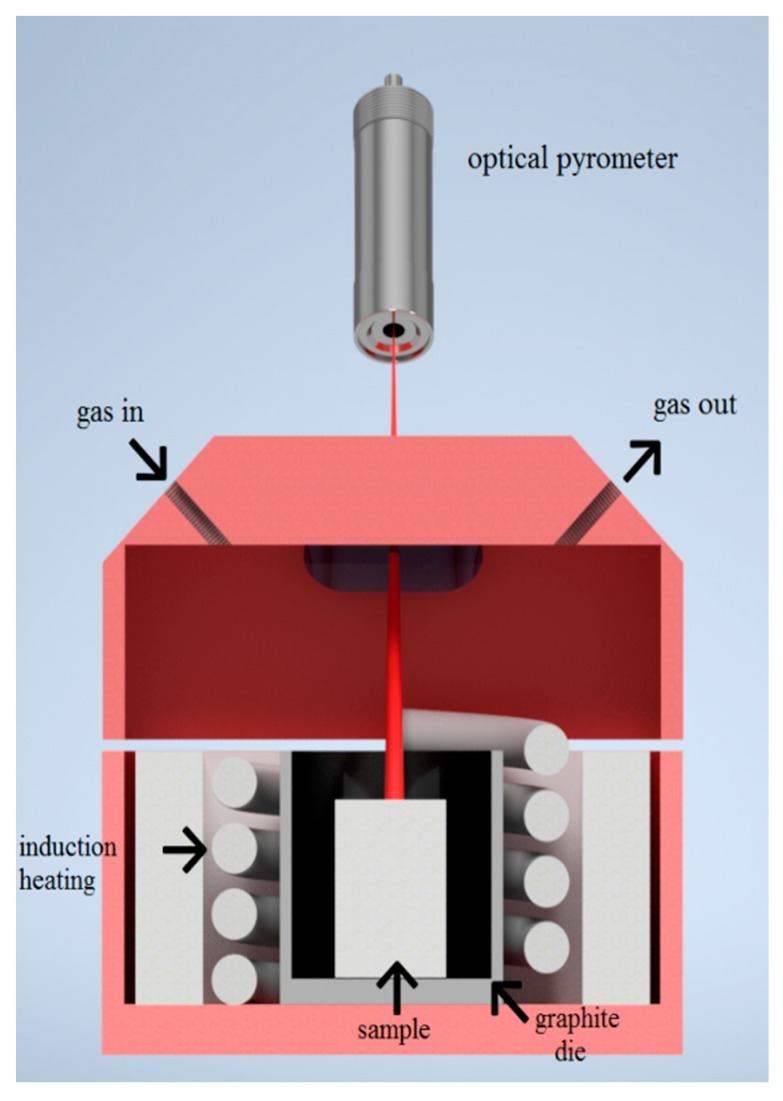
A schematic illustration of the experimental set-up of induction heating.

Samples were prepared from elementary powders (titanium: purity 99.5%, particle size 44 µm; and aluminum: purity 99.62%, particle size 44 µm, STREM CHEMICALS, Newburyport, MA, USA). The powders were mixed to 3 g of the mixture and homogenized manually for 5 min. The powder mixture with the chemical composition TiAl63 (in wt.%) corresponded to the TiAl_3_ phase [17]. Subsequently, 3 g of the mixture was compacted into a green body by the universal loading machine, LabTest 5.250SP1-VM (Labortech, Opava, Czech Republic), at pressure 450 MPa for 5 min. The obtained samples had a diameter of 10 mm. Reactive sintering was performed in an induction furnace (Leybold-Heraeus GmbH, Cologne, Germany), where heating was observed and recorded by an optical pyrometer, Optris OPTP20-2M (Optris, Portsmouth, NH, USA), under Ar atmosphere. Various heating rates were studied, and their values were determined according to the slope of the obtained curves: 19, 59, 89, and 102 °C/min. The scheme of the used apparatus is illustrated in Figure 10. This method enabled us to perform a thermal analysis at a much higher heating rate than during a common, differential thermal analysis. In order to find if the phases formed below the melting point of aluminum, green bodies were inserted into silica ampoules and annealed for 8, 24, and 48 h at 450, 500, and 600 °C, respectively.

Reactive-sintered samples were ground by sandpapers with SiC abrasive particles (P80–P2500, Hermes Schleifmittel GmbH, Hamburg, Germany), and polished by suspension Eposil F (ATM GmbH, Mammelzen, Germany) mixed with hydrogen peroxide in volume ration 1:6. Kroll’s reagent (5 mL HNO_3_, 10 mL HF, and 85 mL H_2_O) was used for etching. The microstructure was observed by the scanning electron microscope, TESCAN VEGA 3 LMU (Tescan, Brno, Czech Republic), equipped with the OXFORD Instrument, X-max 20 mm^2^ SDD EDS analyzer (Oxford Instruments, High Wycombe, UK) for the identification of the chemical composition of the individual phases. The phase composition was studied by X-ray diffraction using the PANalytical X′pert Pro software package with the PDF2 database (PANalytical, Almeo, The Netherlands).

## 5. Conclusions

The present results show that the reactions in the TiAl63 (in wt.%) starting powder mixture proceed in two stages. The solid-state process occurs below the melting point of aluminum, leading to the preferential formation of the Ti_2_Al_5_ phase already at 450 °C. The TiAl_3_ phase formed consequently by the reaction between this phase with aluminum. During rapid heating in induction furnace, this solid-state reaction stage is skipped and the self-propagating high-temperature synthesis reaction is initiated at the interface between solid titanium and molten aluminum, i.e., above the melting point of aluminum. At this stage, the TiAl_3_ phase forms as the main product of sintering, accompanied by the other aluminum-rich phase, Ti_2_Al_5_. The Ti_3_Al phase formed only around unreacted particles of titanium, and its formation was probably enabled thanks to the reaction between titanium and an already present phase, probably the TiAl_3_ phase, or thanks to the high temperature and shorter time of reaction.

## Figures and Tables

**Table 1:** Reaction temperatures.

Heating Rates (°C/min)	T_onset_ (°C)	T_maximum_ (°C)	T_offset_ (°C)
19	662	705	680
59	735	770	778
89	777	817	828
102	791	833	848

**Table 2:** Chemical composition of the Ti_3_Al area (EDS).

Heating Rate (°C/min)	Ti (wt.%)	Al (wt.%)
59	78.1 ± 0.8	21.9 ± 0.8
89	77.3 ± 0.7	22.7 ± 0.7
102	77.1 ± 0.5	22.9 ± 0.5

**Table 3:** Chemical composition of the Al area found in the microstructure annealed at 600 °C for 24 h (EDS).

	Ti (wt. %)	Al (wt. %)
Al area	6.6 ± 3.9	93.4 ± 3.9
